# Communities of practice for supporting health systems change: a missed opportunity

**DOI:** 10.1186/s12961-015-0023-x

**Published:** 2015-07-25

**Authors:** Anita Kothari, Jennifer A Boyko, James Conklin, Paul Stolee, Shannon L Sibbald

**Affiliations:** Western University, School of Health Studies and Schulich Interfaculty Program in Public Health, 1151 Richmond St, London, N6A 3K7 Canada; Western University, School of Health Studies and Faculty of Information and Media Studies, London, Canada; Department of Applied Human Sciences and Élisabeth Bruyère Research Institute, Concordia University, Portland, USA; University of Waterloo, School of Public Health and Health Systems, Waterloo, Canada; Western University, School of Health Studies, Schulich Interfaculty Program in Public Health and Department of Family Medicine, London, Canada

**Keywords:** Communities of practice, Health systems, Knowledge translation, Long-term care

## Abstract

**Background:**

Communities of practice (CoPs) have been used in the health sector to support professional practice change. However, little is known about how CoPs might be used to influence a system that requires change at and across various levels (i.e. front line care, organizational, governmental). In this paper we examine the experience of a CoP in the Canadian province of Ontario as it engages in improving the care of seniors. Our aim is to shed light on using CoPs to facilitate systems change.

**Methods:**

This paper draws on year one findings of a larger multiple case study that is aiming to increase understanding of knowledge translation processes mobilized through CoPs. In this paper we strategically report on one case to illustrate a critical example of a CoP trying to effect systems change. Primary data included semi-structured interviews with CoP members (n = 8), field notes from five planning meetings, and relevant background documents. Data analysis included deductive coding (i.e. pre-determined codes aligned with the larger project) and inductive coding which allowed codes and themes to emerge. A thorough description of the case was prepared using all the coded data.

**Results:**

The CoP recognized a need to support health professionals (nurses, dentists) and related paraprofessionals with knowledge, experience, and resources to appropriately address their clients’ oral health care needs. Accordingly, the CoP led a knowledge-to-action initiative that involved a seven-part webinar series meant to transfer step-by-step, skill-based knowledge through live and archived webinars. Although the core planning team functioned effectively to develop the webinars, the CoP was challenged by organizational and long-term care sector cultures, as well as governmental structures within the broader health context.

**Conclusion:**

The provincial CoP functioned as an incubator that brought together best practices, research, experiences, a reflective learning cycle, and passionate champions. Nevertheless, the CoP’s efforts to stimulate practice changes were met with broader resistance. Research about how to use CoPs to influence health systems change is needed given that CoPs are being tasked with this goal.

**Electronic supplementary material:**

The online version of this article (doi:10.1186/s12961-015-0023-x) contains supplementary material, which is available to authorized users.

## Background

Communities of practice (CoPs) are “groups of people who share a concern, a set of problems, or a passion about a topic, and who deepen their knowledge and expertise in this area by interacting on an ongoing basis” [1]. CoP members apply and heighten their skills and knowledge about a common topic area through formal and informal interactions [2,3]. The concept can be traced back to Lave and Wenger [4], who, drawing on adult learning principles, argued that knowledge and learning develop in specific contexts, implying that social relationships in a relevant setting are more important than the classroom for professional development purposes. This type of learning community represents a more equal relationship between experts and learners, as opposed to the more traditional approach of learning between teachers and students [2]. In addition, CoPs can support mutual consultations that help shape individual members’ professional identities [5].

CoPs have been used to support health care practice, but there remains a weak understanding, particularly in terms of formal and informal structures, of how CoPs might be used as a mechanism for supporting change across different organizations and at different levels of the system (from front line care to governmental policy). Kitson [6] reflects on this shortcoming in the context of the knowledge translation (KT) literature, and suggests that the prevailing linear, rational view of the uptake of knowledge has been unhelpful for widespread adoption and change. In contrast, she describes the innovation perspective of Van de Ven et al. [7], where “…new ideas are developed and implemented by people who engage in relationships with others and make adjustments needed to achieve desired outcomes within an institutional and organizational context” [6]. This perspective draws from non-medical fields, such as sociology and action science, where knowledge is seen as part of a systemic social process consisting of knowing, acting, and structuring [8]. Given that CoP structures allow members to draw on experience, reflect on action, and make adjustments after feedback on action [6], we suggest that CoPs are social contexts that have the potential to drive systems change. This process can lead to the questioning of basic assumptions that underlie current policies, practices, and programs [6], which in turn can lead to systems improvements. This might be done systematically by using CoPs as the facilitator of change at various levels of a system supported by innovations (for best practices, for example) and other contextual knowledge. However, how to engage CoPs with a complex system has not yet been the subject of extensive attention by scholars.

Leveraging CoPs to achieve various functions and outcomes has been examined across sectors. CoPs in business and health sectors have promoted knowledge sharing, knowledge creation, and identity building [3]. CoPs are also seen as a knowledge management strategy that organizations can use to support innovation [2], in part because participation of all members is encouraged and CoPs support a social structure that is based on mutual respect, trust, and information sharing [2]. For example, Iaquinto et al. [9] found that purposefully designed CoPs supported collaboration and knowledge sharing across disciplinary and divisional boundaries in a state-level government department. Organizational leaders understand that CoPs can facilitate the sharing of explicit and tacit knowledge, and can be used to link learning with performance [10].

In the health sector, CoPs are also being used as a strategy to support KT, which involves dissemination, exchange, and application of knowledge to efficiently and effectively strengthen health systems and improve people’s quality of life [11,12]. It includes multiple steps, from the creation of new information through high-quality research to the application of knowledge and innovation to yield beneficial outcomes [11,12]. Advances in knowledge can take years to be implemented into practice, pointing to a knowledge gap in the interim [12]. As a result, KT aims to increase knowledge utilization and ensure that the best available evidence is used to inform policy and practice.

The available literature about CoPs and KT focusses largely on their use within organizational, clinical, or community contexts. Health care organizations concerned with improving the use of research in practice have used CoPs as a way to support KT through the implementation of practice improvements (e.g. clinical practice guidelines use by physicians) [13,14]. A recent systematic review by Mairs et al. [15] found that online or virtual CoPs are a key facilitator of KT in the health care field. Gagnon [16] also reminds us that CoPs can facilitate a more integrated approach to KT by supporting active collaboration and exchange between researchers and knowledge users throughout the research process [16]. The social learning that occurs within CoPs has been shown to be facilitated by knowledge brokers who engage in relational, analytical, and technical activities that help people work on common concerns and challenges [17]. CoPs are being used to support collaboration across national or international jurisdictions for improved knowledge generation and dissemination [14,18], by different types of health practitioners [19,20], and for health policy development to promote, for example, public health financing practices in low-income countries transnationally [21]. Uncovering the mechanisms that contribute to their success, such as how CoPs interact with formal structures within and across health organizations and how organizations leverage their access to such communities to build their internal knowledge assets, is important for advancing the field [16,22].

In this paper, we examine the experience of a CoP in the province of Ontario, Canada, as it engages in KT efforts to improve practice related to the care of seniors. By selecting a case that is strategically important, our purpose is to problematize the CoP’s ability to reach its full potential, and to demonstrate that a gap in the literature exists regarding the provision of theoretical and applied direction for affecting systems change. First, we describe the case, followed by a brief description of the problem that the CoP was trying to address. Then, we provide results from our thematic analysis and discuss these findings by building on the work of Kitson [6], which describes the purposeful integration of systems theory with KT to enable more expedient uptake of knowledge into practice.

### Research setting

When this study was conducted, the Seniors Health Research Transfer Network (now the Seniors Health Knowledge Network (SHKN)) was a large network that worked to improve the delivery of health care for Ontario seniors by facilitating KT among health practitioners [23]. SHKN promotes KT through a library service, knowledge brokers, local implementation teams, collaborative technology, and CoPs. The more than 8000 CoP members identify innovations, translate evidence, and implement changes in health settings to improve seniors’ health [24]. Since its launch in 2005, SHKN has become a significant knowledge transfer network linking Ontario caregivers, policymakers, and researchers who focus on improving the care of seniors.

## Methods

This paper draws on findings from a larger, 3-year multiple case study [25] with the broad aim of increasing our understanding of the KT processes mobilized through CoPs working to improve practice and the health of Ontario seniors. More specifically, this paper focusses on one of the nine cases that comprise the overall study. Case study methodology provides a deep understanding of a situated phenomenon such that the reader’s experience of that phenomenon is enriched [26]. In this paper, we report on unique findings related to the research question – What KT processes are initiated through the CoPs? – that led us to contemplate the role of CoPs in systems change. The Health Sciences Research Ethics Board of Western University (#17879E), Bruyère Continuing Care Research Ethics Board (# M16-11-004), Concordia University Human Research Ethics Committee (# HU2010-115), and University of Waterloo Office of Research Ethics (ORE # 16894) approved the project.

### Sampling

Purposive sampling was used to select SHKN CoPs that had a clear KT objective, represented diversity across cases, and had the potential to promote learning about KT processes in CoPs in relation to a variety of frontline contexts [25]. In this paper, we strategically focus [27] on one CoP arising from the first year of data collection. We do so because in analysing the activities, persons, places, and resources involved in this particular situation, it became clear that it provided a critical example of a CoP’s experiences in trying to affect systems change, from which there was something to learn if we are to move forward on this front [28]. We report on an evocative example of a case selected for its explicit attempt to invoke broad, sustainable change rather than change in a few institutions or through developing practice tools, in the hopes of raising questions and stimulating debate about the realities of CoPs as a mechanism for health systems change [27].

### Data collection

Data were collected using semi-structured interviews, observation, and document review over a year time period. A detailed description of the data collection procedures is available from the published research protocol [25]. First, four semi-structured, 30 minute contexting interviews were conducted with CoP leaders at the start of the observation period to learn about the CoP’s objectives, who was involved, what activities would occur, when they would occur, what knowledge or evidence was being assembled and from where, what organizational contexts might receive the knowledge, and what facilitative mechanisms would be used. The CoP leaders were selected based on their active involvement in the CoP, including significant roles in forming and maintaining the CoP. Contextual data resulted in redundant information, i.e. data saturation, by the fourth interview. A research assistant observed (auditorily) all of the teleconferences planning meetings (n = 5) held by the CoP over a 6 month period to plan the KT initiative. The length of time for the meetings ranged from 25 to 80 minutes, and were usually led by the same individual. The number of participants ranged from two to five (in the results we note there was a larger core planning team, from which these participants were drawn). The topics discussed included updates on administration of the CoP (e.g. membership), webinars and resource development (i.e., KT initiative), future planning, and new issues and opportunities. After each meeting, rough field notes and audio recordings were used to develop more formal field notes, which reflected the standard template being used by each case in the larger study. Field notes captured emerging patterns with respect to knowledge types, facilitation and other roles, and contextual factors. Third, relevant documents about the CoP and the KT initiative were requested from the CoP liaison. Fourth, follow-up interviews (i.e. three semi-structured; one informal) were conducted to further understand the behaviours, activities, and environment related to the KT initiative. These interviewees were purposively selected by the research team to include a mix of CoP members with leadership, knowledge brokering, and knowledge user roles (in keeping with the published protocol). Two CoP members participated in both the contexting and follow-up interviews. The interview questions were devised by the primary investigators of the larger case study who have extensive expertise and experience related to qualitative research methods, KT, and the long-term care (LTC) sector in Ontario. Interview questions were based on dimensions of the Promoting Action on Research Implementation in Health Services (PARiHS) framework [29]). The PARiHS framework was selected because of its common usage in the KT field, thereby increasing the transferability of our study findings, and because of its unique focus on facilitation in implementation efforts. Additional file 1: Appendix A contains the interview guides for the contexting and follow-up interviews; knowledge users on the larger research team ensured the questions had face validity.

Each interviewee was given the opportunity to review their transcript in order to correct errors or add information; no substantial changes were requested. All CoP members who participated in the planning meetings and interviews completed an informed consent to take part in the study. Interviews lasted approximately 60 minutes. The semi-structured interviews were audio-recorded and transcribed, and field notes were prepared following the informal interview. All data were stored and analyzed using NVivo 9/10.

### Data analysis

The study involved two phases of data coding. The first phase focussed on analysing all transcripts and field notes using deductive coding [30]. In this phase, researchers used pre-determined codes (developed by the main study’s principal investigators and based on the main study’s overall research questions, so that each case could be compared using cross case analysis) that aimed to 1) answer the research questions; 2) assess if/how evidence, facilitation, and context were reflected in the data (drawing on the PARiHS framework); and, 3) gather information about the background and activities associated with the KT initiatives (Additional file 2: Appendix B details the coding nodes and descriptions). The second coding phase involved more comprehensive inductive coding and analysis of the text, which allowed new themes to emerge from another thorough review of the data [31]. One team member carried out all the deductive and inductive coding, which was reviewed by a second team member; differences were resolved through discussions involving one of the principal investigators. From here, the team prepared a thick description of the case study that described the knowledge informing the CoP’s KT initiative, the recipients of the knowledge, the knowledge facilitation and translation mechanisms (adaptation, understanding, utilization), and the involvement of users in the KT process [25]. A predetermined format ensured that the case study report included a case narrative based on the deductive coding, interaction maps based on clusters of themes arising from the inductive coding, and answers to the research question based on all data (see [25] for further details). The case study report (which was roughly 60 pages long) was then subject to further interrogation at a face-to-face team meeting to ensure interpretations were reasonable. In this paper, we report on unique results related to the CoP trying to affect systems change; subsequent publications will elaborate on the common patterns arising from the interaction maps in a cross-case analysis.

## Results

### Background

This CoP, formed in 2009, was in its third year of operation at the time of study and was composed of approximately 60 individuals from across Ontario. It aimed to provide evidence-based and clinically relevant information to health professionals who provide oral care to older adults in LTC and hospital settings. The 11 CoP members who participated in our study (i.e. the core planning team) wanted to bring together informal and formal networks of health care and oral health professionals with frontline workers who guide or provide direct care for the frail elderly. The CoP members aimed to do this by raising awareness, providing education and learning opportunities, and by promoting collaboration and networking between the health care and dental care sectors. The CoP planning team included four co-leads, one knowledge broker, and one librarian. The core planning team held meetings to plan how to refine their process for creating the KT initiative, delivering it, and then appropriately archiving it for ongoing access by the target population. This case study focused on the planning, implementation, and evaluation activities of the core planning team related to the KT initiative.

### The practice challenge

The Ontario long-term care sector, although engaged with quality improvement initiatives to enhance care for the elderly, has placed less emphasis on oral health care. As a result, health professionals (e.g. nurses, dentists) and related paraprofessionals lack the knowledge, experience, and resources to appropriately address the oral health care needs of their elderly clients.

### The case

The case, or KT initiative of this CoP, was a seven-part webinar series meant to transfer step-by-step skill-based knowledge through live and archived webinars. This series was built on the success of a previously developed series focused on basic oral care skills for the elderly. The purpose of the current initiative was to support practice-based skills in oral health in a specific population (stroke) and oral health condition (halitosis). The target audience for this initiative was frontline health professionals who provide care for older adults in LTC and hospital settings. The CoP identified the information for their initiative from a variety of places including best practice guidelines and synthesized research. Experts were also called on from the health and dental fields to aid in resource development. The research was repackaged into manageable and actionable learning segments that were viewed in real-time, interactive sessions or asynchronously via web-based archives and portable data devices. Each webinar was less than 15 minutes in length, with many images, directive words, and simple language. The live webinars incorporated a question and answer format at the end, resulting in a total time of the live webinars of 30 minutes. Each of the seven webinars was offered three times – the first two were trials, used to create questions for the final polished version (to be archived on a website). The goal was to post the final version of all seven webinars within 30 days.

### The CoP’s experience in approaching the challenge

In this section we first briefly describe how the core planning team functioned internally to develop the webinars. Then, we turn the lens outwards and discuss how the CoP was challenged by organizational and LTC cultures within the broader context of health system and governmental structures.

#### Internal functioning

The group was officially designated a CoP by the larger SHKN network. In addition, the functioning of the 60-member group exhibited several CoP characteristics identified in the literature [7]. For example, identity in the CoP was supported by sign-up through a website (i.e. commitment), which clearly outlines the focus on the practice topic (i.e. a domain of interest) without restrictions on residency or professional background. Not only can members access resources like reading lists and newsletters but they can also participate virtually by interacting with colleagues in a members-only web area, indicating the value placed on members’ competence. Facilitating structures, such as live webinar technology and workshops, promoted discussions and joint learning. As described by one participant, “*…I think we did 15 or 17 presentations, like one on one with staff mostly, like one on one, one to three ratio, so really good high levels and pretty intense interactions*”. At the time of study, the 18-month work plan identified the following objectives: 1) to further develop the CoP through targeted growth and activity reviewing; 2) to undertake a membership evaluation survey and review data; 3) to develop and deliver a series of webinars on oral health skills; and 4) to archive oral health skills webinars and resources. As one participant said, “*The purpose of the initiative is for people from across the province to work together to help improve oral care in residents in long term care in Ontario.*” In this way the CoP continued to build a shared repertoire.

The case study revealed some evidence that suggests the core planning team functioned effectively. First, it included highly experienced health care professionals interested in dental and oral health who provided extensive content expertise. Their experiences, and their previous connections in the wider community, were vital to the group’s success. For example, one CoP leader persuaded an external organization to host the webinar series on its organizational websites for practitioners to access at their own convenience. Second, the diverse composition of the planning team was constantly refreshed through an evolving membership. New members brought innovative ideas and approaches. Despite this evolving membership, the common drive to improve oral health care among the frail elderly allowed for an ongoing common identity and clear vision. New members continued to draw on their own web of connections such that, over time, the CoP became a network of networks. Third, participants indicated that they were part of a positive collaboration. They spoke about sharing their brainstorming ideas with each other by email, indicating a level of mutual comfort. They praised each other (“*she’s outstanding at taking the huge amounts of content that exists in any topic area and reducing it to its nuts and bolts…*”, “*I think everybody’s open, everybody’s very creative and everybody’s always looking for solutions*”) and they spoke about what makes the team function well (“*…so it’s worked really well so we sort of mutually challenge each other*”).

Fourth, the CoP managed their workload by building on their past accomplishments. In particular, when they created their webinars, they made an important decision to focus on topics they had previously addressed. This “recycling” of ideas was less taxing on the group than starting with new topics. As one respondent explained:

“*…I’ve done several presentations to the stroke audience on oral health so I’ll pull from that, from those slides that I’ve already done and take some of that information and put it in there. We’ve done dementia presentations several times and end of life care … and* [person’s name] *has done stuff on dry mouth and* [person’s name] *has done stuff on bad breath, so it helps if you’ve got something you can also pool from.*”

Fifth, the CoP recognized the need to tailor the content of the webinars to the intended audience. This involved careful consideration of the message, the audience, and the communication infrastructure. In terms of the message, the CoP used both explicit knowledge (such as research findings and best-practice guidelines) and tacit knowledge in their webinars. Knowledge derived from research and best practice guidelines was supplemented with skills-based knowledge (“*some of it comes from research guidelines, some of it comes* [from] *a solid clinical knowledge base that exists there*”). This tacit knowledge was communicated by providing a video demonstration of the techniques required for proper oral health. The CoP hoped that by demonstrating the actual skills, they could translate a message that is difficult to articulate using observed action. This tailoring of content was also evident in the way the webinars were packaged in multiple modalities (e.g. a live webinar with a question and answer session, a podcast, an archived webinar available on the internet, and a recorded webinar available on DVD). Further, the content was tailored to specific groups. For example, the webinar information was repackaged to speak more easily to professionals who are more disease-oriented (e.g. stroke) than technique oriented. The webinars were also ‘beta-tested’ by encouraging CoP members to test webinar components in their own institutions. Based on testing, the core planning team received feedback on the effective and ineffective aspects of the products as well as suggestions for improvements. Once the beta-testing showed that the approach was successful at the test sites, the webinars were delivered to the broader LTC community.

#### Contextual challenges

Study participants indicated that the main challenges encountered by this initiative had to do with the culture of LTC organizations and the structure (regulations, procedures) of the broader LTC sector. Clearly, these two forces interact, sometimes making it difficult to determine if common observations across organizations were indeed cultural in nature (i.e. reflecting a shared culture in the LTC sector) or instead were structure-related (i.e. reflecting policies). At the time of this case study, it was evident that most CoP members felt that, to implement the oral health care changes that were favoured by the CoP, it would be necessary to influence and change the culture of LTC. It was less evident that study participants saw a need to influence health care delivery priorities by pursing changes to provincial policies.

There is an awareness that those on the front-line who are providing oral health care need more training (both while in school and through continuing professional development) as well as more support from their organizations’ leadership. In most organizations, these seemed to be lacking. The CoP was working to create a culture of awareness and support from the bottom up. The challenge for this CoP seemed to be about finding a way to reach management, even though their primary audience has typically been on the front-lines of care. One CoP member described this challenge as follows:

“*One of our biggest barriers is trying to figure out why staff won’t do oral care, and they feel quite justified in not doing oral care, yea if you say to them would you just ignore pericare and not do that? Well, no, they wouldn’t ignore that. But they, they are quite happy to ignore the oral care.*”

Oral health is not seen as a geriatric health care concern in the larger context. When speaking about managers in LTC homes, for example, one participant noted that:

“*It’s so hard to reach these guys and when you’ve got something that is just not seen as being really important, … because they won’t sit down long enough* [for us] *to explain that, so it’s just, so it’s getting that* [message] *out that’s really so important.*”

Without leadership support it appears difficult to find resources or committed individuals who are willing to push the issue forward in an organization. As one participant noted “*it is hard to rally the troops around this issue*”, since there are currently so many other informal and mandatory training sessions required by staff on topics such as drug safety training and order entries. The lack of mandatory training around oral health suggests that the issue is a low priority for organizations or government. As one study participant put it: “*But the challenge we have in hospital is there’s always so many learning needs and opportunities out there competing for staff time when they have so very limited time…*”. Similarly, another participant explains:

“*It’s tough and it’s getting a whole lot worse, … there’s so many things they* [staff] *have to do as mandatory education, … it’s just one thing after another for people who have no time in their day, so then, and this is not to minimize it in any way, but then to say hey, why don’t you come to an in-service on oral care, like it does seem almost preposterous really, ….this* [oral care] *is really important stuff,* [staff] *need to make sound decisions about what they become involved in or not, because it’s a hospital thing, you know, this* [other training] *is mandatory, you must* [attend], *so by the time those things get taken care of, there is next to no time left for what people would consider the nice to knows, people themselves think of them as need to knows. But you know some of the things that we value just don’t get high enough up on the radar.*”

The quote above suggests that the organizational culture values high-quality care, and thus they are open to disseminating information on improved oral health care. However, the culture also values mandates, rules, and procedures, and hence front-line staff and managers focus on numerous competing priorities. As a result, not taking effective action on oral health care might be due to its absence in official organizational or government policy.

The CoP is at a stage where it can consider feedback and evaluations about their knowledge products and webinars with the future in mind. At the time of this study, the CoP had just started branding their product to achieve greater recognition and legitimacy. The CoP understands that changing organizational culture to address oral health will require incremental shifts in attitudes as well as developing organizational support tools (e.g. templates for oral health policies that organizations can adapt). Nevertheless, how to deliberately change organizational cultures through attitudes, support tools, and other mechanisms seems elusive at the moment. For example, one participant explains:

“*Well I think, from my perspective, in addition to awareness raising, I think we’re probably a little bit beyond that, and awareness about oral health being an issue, but I think what we’re trying to do is extinguish some old practices and raise awareness and try to help people to see what the evidence is on some of the practices that haven’t been part of nurses’ basic care practices, and try to really kind of push those, so many people are talking about and they think it’s kind of commonplace and it becomes ingrained in current practice, so I think that that’s part of what we’re trying to achieve.*”

The CoP did not seem to consider advocating for changes to governmental or LTC sector structures to support their current front-line efforts or future organizational cultural targets even though they recognized that getting leaders, who respond to legislative priorities, onside with the oral health care agenda was crucial for change.

## Discussion

This case study highlights the experiences of a CoP focused on using evidence to change provincial practices by health and oral care practitioners working with the elderly. A webinar series was developed by the CoP as its KT initiative. A strength was that CoP membership reflected the target audience, providing a close understanding of the gap in service provision and the feasibility of potential solutions. To develop the webinars, the CoP informally assessed the needs of stakeholders, created links through networks and across disciplines, and called upon internal and external experts, utilizing resources reflecting experiential and scientific knowledge. The CoP depended on a variety of webinar modalities and tailoring of final products to meet the needs of knowledge users. It is likely that the CoP was able to accomplish this due to its collaborative internal functioning. This CoP exhibited the features of a successful CoP described by others [21,29].

The dynamics of the CoP influenced specific KT processes. The CoP was composed of a diverse membership representing relevant locations, like LTC settings, and the intended audience of the KT initiative. Members played a huge role in bringing evidence into the discussion about knowledge creation. For example, CoP members brought evidence in the form of guidelines and best practices. In addition, concepts around knowledge generation were evident throughout the email communications and planning meetings. In terms of adapting the KT initiative, some CoP members were front-line clinicians who introduced the webinar straight to the end users within their work context, and then provided feedback on what worked and what did not. The CoP exhibited a culture of passion, hard work, and dedication to the cause. The CoP dynamics (the people involved and the collective drive of the group) were a significant enabler to the development and adaptation of the successful KT initiative. Throughout the planning of the KT initiative, there was awareness of a collective commitment to achieving a successful CoP.

However, the CoP met with resistance externally, from LTC organizations generally, despite the need for oral health knowledge. Support from management was lacking. Staff time is directed to those professional development opportunities that are legislatively mandated, which currently does not include oral health care sessions, perhaps reflecting a larger disengagement with oral health care in the sector.

Recent reviews of the CoP literature [3,10,33] provide some guidance to make sense of what was observed in this case study. In previous studies, practitioner change was enacted through practitioner involvement as a member of a CoP (e.g., [13]), but examples of influence beyond CoP members were few. Some studies have reported on CoPs and their broad impact on systems where CoPs were part of a multi-faceted intervention [32-36]. One study in particular helped clarify our thinking about the findings from our own problematic CoP; Taplin et al. [37] developed a regional plan to increase cancer screening rates using a multi-faceted approach that included a regional and three local CoPs. The local CoPs, comprised of hospital and community partners, were tasked with sharing ideas, developing approaches, and encouraging local action, similar to our case study CoP. Their collaborative learning strategy led to increased screening.

The Taplin study made us realize that CoPs should be re-conceptualized to acknowledge that one or a few CoPs in a sector may not be enough to affect longer term, sustainable practice change that will lead to real impact on our health system. Two intersecting avenues are worth considering if systems improvements are a goal. First, as demonstrated in previous studies, CoPs, as part of a larger intervention, show promise. The second avenue, which was identified directly from our study findings, is the need to use CoPs to affect larger cultural understandings about the relevance and importance of a practice. The intersection of these avenues suggests that sustainable systems change requires small-scale practice change, mid-level organizational change, and large-scale policy change. In Ontario, SHKN is using CoPs as a KT strategy by improving the capacity of health and social service organizations to use research evidence. It is anticipated that the overall findings from the 3-year study of these CoPs (which the current study is part of) will elucidate concepts and practical issues involved in using CoPs to support systems transformation. The current CoP literature does not currently take up these issues deeply, probably owing to the fact that most studies to date do not articulate sector-wide change as a prime focus. Nevertheless, the CoPs in the health arena are being co-opted for this wider purpose.

As per systems theory and building on Kitson’s work [6], we suggest supporting change at multiple levels (micro, meso, and macro) of the system and offer some thoughts about the appropriate scope of the system. In other words, what is the scale at which a solution might be attempted? Scholars are sometimes guilty of simply equating the “context” with the “system”, but a manageable definition of system is key for improved performance. How do we frame the problem so as to encompass sufficient elements of the relevant system to bring about change? As illustrated by the oral health case, we must consider those practitioners whose behaviour we want to impact, those social structures that shape that behaviour, and then address the assumptions and paradigms that inform that behaviour. Some recent examples point to a regional geographical boundary (in contrast to a province or entire country) as a way to draw an outline around the scope of change. A prime example are the UK’s Collaborations for Leaderships in Applied Health Research and Care, which are regional CoP-like partnerships meant to enable KT, organisational learning, and sustainable change in how applied health research is conducted and implemented in communities [38]. As another example, Bramwell et al. [39], when speaking about university-industry partnerships for innovation and creativity, make a strong argument for the localized nature of KT, suggesting that the “proximity effect” is important for accessing the source of research products (i.e. CoPs) and related tacit or unpublished knowledge. The local collaborations – or CoPs and long-term care institutions, in our case – are characterized as interdependent, multidisciplinary, and multi-sector, and are able to react quickly to dynamic shifts in the setting. This “innovation ecosystem approach” is gaining traction worldwide and requires that CoPs form collaborations with their target institutions and government.

This discussion assumes that CoPs are an appropriate KT innovation by which to influence change at a systems level; this assumption is based on recent policy directions promoting CoPs for this purpose [40,41]. The CoP as a social interaction process was successful in achieving its primary objective related to KT: developing, disseminating, and archiving its webinar series. It could be the case, however, that the subsequent resistance to practice changes across the LTC system, which was related to cultural norms and structural priorities, might have been expressed regardless of the KT innovation used. Nonetheless, governments and organizations are promoting and using CoPs as an important mechanism by which to invoke systems change in the health sector. Because CoPs do not arise from a specific organization, but rather span a variety of institutions from across the health system, it is possible that CoPs are well-positioned to undertake system-change interventions at several system levels. For this reason alone further research is required to understand the best ways that CoPs can be supported in this change mandate.

## Conclusion

While the findings presented in this paper were limited to observations arising from one knowledge-to-action initiative, the case was strategically selected to shed light on the understudied topic of using CoPs to facilitate systems change broadly. This study contributes to a better understanding of why CoPs may not be able to affect change in the health system. Our findings demonstrated that, in this case, the CoP functioned as an incubator that brought together best practices, research, experiences, a reflective learning cycle, and passionate people. Nevertheless, CoP efforts to stimulate practice changes across the LTC system were met with resistance by cultural norms at the organizational and sector levels, which in turn were influenced by structural priorities. Opportunities to improve care for seniors’ health will be missed unless provincial CoPs are better supported in their efforts to facilitate systems change. The CoP literature does not provide sufficient guidance about how to use CoPs to influence health systems change. Therefore, more primary studies that evaluate the role and influence of CoPs in system-level change are required.

## Additional files

Additional file 1:
**Appendix A: Contexting interview guide.** (PDF 18 kb) Additional file 2:
**Appendix B: NVivo node descriptions for deductive coding.** (PDF 27 kb)

## Abbreviations

CoPs: Communities of practice
KT: Knowledge translation
LTC: Long-term care
PARiHS: Promoting Action on Research Implementation in Health Services
SHKN: Seniors Health Knowledge Network
