# Erratum to: Communities of practice for supporting health systems change: a missed opportunity

**DOI:** 10.1186/s12961-015-0033-8

**Published:** 2015-11-05

**Authors:** Anita Kothari, Jennifer A. Boyko, James Conklin, Paul Stolee, Shannon L. Sibbald

**Affiliations:** Western University, School of Health Studies and Schulich Interfaculty Program in Public Health, 1151 Richmond St, London, N6A 3 K7 Canada; Western University, School of Health Studies and Faculty of Information and Media Studies, London, Canada; Department of Applied Human Sciences and Élisabeth Bruyère Research Institute, Concordia University, Portland, USA; University of Waterloo, School of Public Health and Health Systems, Waterloo, Canada; Western University, School of Health Studies, Schulich Interfaculty Program in Public Health and Department of Family Medicine, London, Canada

## Erratum

After publication of the original article [1] it came to the publisher’s attention that references 32 and 33 were cited in the incorrect positions in the text. These errors have been corrected in the original article.

- Reference 32 was added to the following sentence, “This CoP exhibited the features of a successful CoP described by others [21, 29, 32].”
- Citations in the sentence, “Some studies have reported on CoPs and their broad impact on systems where CoPs were part of a multi-faceted intervention [32-36]” were changed to, “Some studies have reported on CoPs and their broad impact on systems where CoPs were part of a multi-faceted intervention [34-36].”
