# Supplementary material for: Communities of practice for supporting health systems change: a missed opportunity
Source: Health Res Policy Syst. 2015 Jul 25;13:33. doi: 10.1186/s12961-015-0023-x (PMC4515005; doi:10.1186/s12961-015-0023-x)
Supplement: Additional file 1: — Appendix A: Contexting interview guide. (PDF 18 kb) [file 12961_2015_23_MOESM1_ESM.pdf]

Appendix A

Contexting Interview Guide

1. Please describe the KTA process that you would like our research to focus on. [Probe for the following: What is its purpose? What outcomes do you hope to achieve? What has already happened? What activities are planned for the next several months? Who is involved from the CoP? What external stakeholder groups are participating?]

2. Is this work building on any previous CoP activity? For example, does the present KTA initiative represent a follow-up to work you undertook last year? Is its design based on lessons you learned from past experiences?

3. What caused you to undertake this initiative? [Probe for: Did the impetus originate within the CoP, did it originate with frontline stakeholders outside the CoP, or did it originate elsewhere?]

4. Where are you sourcing the knowledge for this KTA initiative? [Probe for: Is the initiative dealing with knowledge generated through scientific research, through practice-based innovation and experience, or both?]

5. Describe the KTA processes that you will use to move this knowledge into frontline practices? [Probe for: Will you use face-to-face interactions or presentations? Will there be a web-based seminar or presentation? Are you offering a one-time activity, or will there be follow-on activities? If there are follow-on activities, what will these involve (discussion events, coaching arrangements, mentoring, evaluation)?]

6. Describe the stakeholders who are participating in your KTA initiative, and the frontline organizational contexts where they work. [Probe for: What is their level of readiness for adopting this knowledge and instituting changes in their practice? How important is this knowledge to them? Will both organizational leaders and frontline service delivery staff be actively involved in your KTA initiative? Will you offer any support for them after they begin to implement the knowledge within their practices? How do you think they will use the knowledge (e.g. to make instrumental changes to day-to-day work routines, or to help themselves and others to understand new information about important work-related issues, or to persuade leaders to support the adoption of new work practices)?

7. We hope to recruit members of frontline practices who participate in your KTA initiative to also participate in our research. May we send an announcement about the research to people who sign up to participate?

Follow Up Interview Guide

1. Please tell me about yourself. What is your job title? [Find out if the participant is a doctor, nurse, social worker, PSW, recreational therapist, etc.] Where do you work? In your organization, what is your role? Who do you work with?

2. Tell me about your participation in this project. How did you become involved? What motivated you to participate?

3. Tell me about what happened. What training or coaching activities did you participate in? Have you completed the training, or is there further work being done?

4. What were you trying to achieve or learn by participating in this project? Why was this information important and relevant for you? Is there a specific problem or issue in your workplace that led you to participate in the project?

5. Was your organization supportive of your participation? Did leaders in your organization ask you to participate? Did people in your organization ask you to share your new knowledge with them? What did you do to share the knowledge you gained through the Webinar and follow-up session?

6. Have you used any of the knowledge or skills that you acquired through the training?

7. Do you believe that your participation in this project has resulted in any beneficial changes in your organization? What changed? How did the change come about? What do you base your answer on?

8. What have been your biggest challenges in attempting to apply the knowledge or skills that you acquired through this project?

9. What are the main factors influencing your attempt to implement these new approaches (positive and negative)? What could reduce the negative factors’ influences, and improve the positive factors’ influences?

10. Do you have any success stories that you could share?
