# Supplementary material for: Communities of practice for supporting health systems change: a missed opportunity
Source: Health Res Policy Syst. 2015 Jul 25;13:33. doi: 10.1186/s12961-015-0023-x (PMC4515005; doi:10.1186/s12961-015-0023-x)
Supplement: Additional file 2: — Appendix B: NVivo node descriptions for deductive coding. (PDF 27 kb) [file 12961_2015_23_MOESM2_ESM.pdf]

Appendix B

NVivo Node Descriptions for Deductive Coding*

**Node Name**	**Node Descriptions**
Background	CoP Members: Descriptions about their job(s) outside of the CoP initiative – what they do, where they work; role in the CoP as a whole; their organization(s), setting or activities; (past or present). Includes all CoP members. Includes members of the Core Team (only if unrelated to the Initiative). Related categorical information may be captured in alternate formats (eg. excel) which specify titles, organization names, etc by unique ID.<br><br>The CoP: Descriptions about the CoP history, how the CoP began and activities that are not part of the CoP initiative that makes up the KTA case.
Framing the Initiative	Purpose: Includes purpose, expected outcomes and rationale.<br>*Relates to Contexting Interview Qs 1a, 1b, 3 and any other data that is relevant over the course of the case. 1a) What is the purpose? 1b) What outcomes do you hope to achieve? 3. What caused you to undertake this initiative?*<br><br>Previous Activities: Previous activities related to the KTA case initiative. May also includes previous activities that are not directly related to the current initiative but contribute to its understanding.<br>*Relates to Contexting Interview Qs. 1c) What has already happened? 2. Is this work building on any previous CoP activity?*<br><br>Activities-Processes: Current activities related to the KTA case initiative including planning, KTE events, frontline, follow-up. Includes Field Note header information on date of event, participants etc.<br>*Relates to Contexting Interview Qs 1d) What activities are planned for the next several months? 5. Describe the KTA processes that you will use to move this knowledge into frontline practices? Describes what this CoP initiative does (including planning).*<br><br>Core Team: Describes the core team of CoP members who are planning or leading the specific KTA initiative. Includes relationships, who does what re: the team, the work that needs to be done, skills, aptitudes, thoughts about what works, thoughts about the team – related to the current initiative only; does not include previous activities.<br>*Relates to Contexting Interview Q 1e) Who is involved from the CoP?*<br><br>Stakeholders: Includes/describes organizations (or individuals representing them) participating in the planning or implementation of the initiative;

	target group/audience or recipients of the initiative; and any frontline contexts (those who are expected to implement the KTA changes or who are expected to be impacted). May include previous activities as an indicator of stakeholder readiness.<br>*Relates to Context Interview Qs 6. Describe the stakeholders who are participating in your KTA initiative and the frontline organizational contexts where they work. 6a) What is their level of readiness for adopting this knowledge and instituting changes in their practice? 6b) How important is this knowledge to them? 6c) Will both organizational leaders and frontline service delivery staff be actively involved in your KTA initiative? 6d) Will you offer any support for them after they begin to implement the knowledge within their practices? & 1f) What external stakeholder groups are participating?*<br><br><u>Links</u>: Includes partners, previous partners, loose associations with organizations, people or other CoPs who are not stakeholders.
PARIHS Framework	<u>Context:</u> Receptiveness to change; physical, social, cultural, structural, system, professional/social network contexts; decision making processes; power and authority; values/beliefs; individual and organizational culture; relationships; teamwork; leadership; evaluation; resources (human, financial). This node should describe Context related to the planning phase, the broader CoP, the Core Team and the KTE events. Includes Knowledge User Involvement (consultation, cooperation, collaboration, empowerment) and Type of Knowledge Use (instrumental, conceptual, symbolic). This node may include Previous Activities when they complement the current context.<br>*Relates to Context Interview Q6e) How do you think they will use the knowledge (e.g. to make instrumental changes to day-to-day work routines, or to help themselves and others to understand new information about important work-related issues, or to persuade leaders to support the adoption of new work practices)?*<br><br><u>Evidence</u>: Descriptions, titles and comments about the knowledge that is used as sources of evidence. Include: research, practice based/clinical experience, client experience and preference, tacit knowledge, experiential knowledge and information from the local context. Type of evidence is interpreted broadly. May include previous meeting notes or data documents if they are being used as evidence for the current case.<br>*Relates to Contexting Interview Q4) Where are you sourcing the knowledge for this KTA initiative?*<br><br><u>Facilitation</u> : Roles, skills and attributes that enable (coerce or impede) others in implementing change; includes level, amount and types of support provided; capacity building/enabling others versus doing for others approaches; task versus process focus/roles; learning approaches; internal versus external facilitators. Skills and attributes can include: project

	management, technical, marketing, critical reflection, giving meaning etc. May include push-pull approaches if the data refers to a person doing the pushing/pulling. Includes approaches used in past activities.
Quotable Quotes	
Research Questions	<u>KTA Processes</u>: 1. Knowledge-to-action processes: a) What KTA processes are initiated through the CoPs? KTA processes are related to the movement of knowledge; they include concrete and conceptual processes, and push or pull approaches. b) How well do the three dimensions (evidence, context, and facilitation) proposed in the Promoting Action on Research Implementation in Health Services (PARIHS) framework describe the emergent patterns of knowledge flow? c) To what extent does KTA involve an interaction between explicit knowledge and tacit knowledge?<br><br><u>Role of Human Agents</u>: 2. The role of human agents (current initiative): a) What roles are evident among those who participate in these processes? b) How does the active involvement of knowledge users in the KTA process influence knowledge utilization? c) What human factors support or hinder effective involvement in KTA processes?<br>This node may include push-pull approaches if the data refers to a person doing the pushing/pulling. This node does NOT include past activities – just the current role of human agents?
Technology	References to technology used, how it's used, applications, qualitative descriptions of use, how it facilitates or hinders KTA, etc.
Emerging Nodes	

*This framework was used to analyze data across all three study sites for nine separate case studies. In this paper we report on the unique findings related to one case.
